# Plasma Levels of Endocannabinoids and Their Analogues Are Related to Specific Fecal Bacterial Genera in Young Adults: Role in Gut Barrier Integrity

**DOI:** 10.3390/nu14102143

**Published:** 2022-05-20

**Authors:** Lourdes Ortiz-Alvarez, Huiwen Xu, Xinyu Di, Isabelle Kohler, Francisco J. Osuna-Prieto, Francisco M. Acosta, Ramiro Vilchez-Vargas, Alexander Link, Julio Plaza-Díaz, Mario van der Stelt, Thomas Hankemeier, Mercedes Clemente-Postigo, Francisco J. Tinahones, Angel Gil, Patrick C. N. Rensen, Jonatan R. Ruiz, Borja Martinez-Tellez

**Affiliations:** 1 PROFITH (PROmoting FITness and Health through Physical Activity) Research Group, Sport and Health University Research Institute (iMUDS), University of Granada, 18071 Granada, Spain; lortizalvarez7@ugr.es (L.O.-A.); elihuixu@gmail.com (H.X.); fj.osuna.prieto@gmail.com (F.J.O.-P.); franciscoacosta838@gmail.com (F.M.A.); b.martinez-tellez@lumc.nl (B.M.-T.); 2 Department of Biochemistry and Molecular Biology II, School of Pharmacy, University of Granada, 18071 Granada, Spain; jrplaza@ugr.es (J.P.-D.); agil@ugr.es (A.G.); 3 Leiden Academic Centre for Drug Research, Division of Systems Biomedicine and Pharmacology, Leiden University, 2300 Leiden, The Netherlands; x.di@lacdr.leidenuniv.nl; 4 Division of BioAnalytical Chemistry, Institute of Molecular and Life Sciences (AIMMS), Vrije Universiteit Amsterdam, 1081 Amsterdam, The Netherlands; i.kohler@vu.nl; 5 Center for Analytical Sciences Amsterdam, 1098 Amsterdam, The Netherlands; 6 Center for Biomedical Research, Department of Analytical Chemistry, Institute of Nutrition and Food Technology, University of Granada, 18071 Granada, Spain; 7 Research and Development of Functional Food Center (CIDAF), Health Sciences Technology Park, 18071 Granada, Spain; 8 Turku PET Centre, University of Turku, 20014 Turku, Finland; 9 Turku PET Centre, Turku University Hospital, 20521 Turku, Finland; 10 InFLAMES Research Flagship Centre, University of Turku, 20014 Turku, Finland; 11 Department of Gastroenterology, Hepatology and Infectious Diseases, Otto von Guericke University Magdeburg, 39106 Magdeburg, Germany; ramiro.vilchez@med.ovgu.de (R.V.-V.); alexander.link@med.ovgu.de (A.L.); 12 Children’s Hospital of Eastern Ontario Research Institute, Ottawa, ON K1H 8L1, Canada; 13 Department of Molecular Physiology, Leiden Institute of Chemistry, Leiden University, 2300 Leiden, The Netherlands; m.van.der.stelt@chem.leidenuniv.nl; 14 Leiden Academic Centre for Drug Research (LACDR), Department of Systems Biomedicine and Pharmacology, Leiden University, 2300 Leiden, The Netherlands; hankemeier@lacdr.leidenuniv.nl; 15 Department of Cell Biology, Physiology and Immunology, Maimónides Biomedical Research Institute of Córdoba (IMIBIC), Reina Sofia University Hospital, University of Córdoba, 14004 Córdoba, Spain; mer.cp@hotmail.com; 16 Unidad de Gestión Clínica Endocrinología y Nutrición, Instituto de Investigación Biomédica de Málaga-IBIMA, Hospital Universitario Virgen de la Victoria, Universidad de Málaga, 29016 Malaga, Spain; fjtinahones@uma.es; 17 Centro de Investigación Biomédica En Red (CIBER), Fisiopatología de la Obesidad y Nutrición (CIBEROBN), Instituto de Salud Carlos III (ISCIII), 28029 Malaga, Spain; 18 Biomedical Research Center, Institute of Nutrition and Food Technology “José Mataix”, University of Granada, Parque Tecnológico Ciencias de la Salud, 18071 Granada, Spain; 19 Instituto de Investigación Biosanitaria, 18014 Granada, Spain; 20 Einthoven Laboratory for Experimental Vascular Medicine, Department of Medicine, Division of Endocrinology, Leiden University Medical Center, 2300 Leiden, The Netherlands; p.c.n.rensen@lumc.nl; 21 Department of Physical and Sports Education, School of Sports Science, University of Granada, 18071 Granada, Spain; 22 CERNEP Research Center, Department of Education, Faculty of Education Sciences and SPORT Research Group (CTS-1024), University of Almería, 04120 Almeria, Spain

**Keywords:** endocannabinoid system, gut barrier, gastrointestinal microbiome, inflammation, short-chain fatty acids

## Abstract

Objective: To investigate the association of plasma levels of endocannabinoids with fecal microbiota. Methods: Plasma levels of endocannabinoids, anandamide (AEA) and 2-arachidonoylglycerol (2-AG), as well as their eleven analogues, and arachidonic acid (AA), were measured using liquid chromatography-tandem mass spectrometry in 92 young adults. DNA extracted from stool samples was analyzed using 16S rRNA gene sequencing. Lipopolysaccharide levels were measured in plasma samples. Results: Plasma levels of endocannabinoids and their analogues were not related to beta or alpha diversity indexes. Plasma levels of AEA and related N-acylethanolamines correlated positively with the relative abundance of *Faecalibacterium* genus (all rho ≥ 0.26, *p* ≤ 0.012) and *Akkermansia* genus (all rho ≥ 0.22, *p* ≤ 0.036), and negatively with the relative abundance of *Bilophila* genus (all rho ≤ −0.23, *p* ≤ 0.031). Moreover, plasma levels of 2-AG and other acylglycerols correlated positively with the relative abundance of *Parasutterella* (all rho ≥ 0.24, *p* ≤ 0.020) and *Odoribacter* genera (all rho ≥ 0.27, *p* ≤ 0.011), and negatively with the relative abundance of *Prevotella* genus (all rho ≤ −0.24, *p* ≤ 0.023). In participants with high lipopolysaccharide values, the plasma levels of AEA and related N-acylethanolamines, as well as AA and 2-AG, were negatively correlated with plasma levels of lipopolysaccharide (all rho ≤ −0.24, *p* ≤ 0.020). Conclusion: Plasma levels of endocannabinoids and their analogues are correlated to specific fecal bacterial genera involved in maintaining gut barrier integrity in young adults. This suggests that plasma levels of endocannabinoids and their analogues may play a role in the gut barrier integrity in young adults.

## 1. Introduction

The endocannabinoid system is mainly composed of cannabinoid receptors 1 and 2 (CB_1_ and CB_2_), several endogenous lipids called endocannabinoids, and metabolic enzymes involved in their synthesis and degradation [1]. Anandamide (AEA), a member of the N-acylethanolamines class, and 2-arachidonoylglycerol (2-AG), as part of the acylglycerol class, are the two primary endocannabinoids [2]. Structural analogues of these two endocannabinoids, as well as arachidonic acid (AA), which is the downstream metabolite of the primary endocannabinoids AEA and 2-AG, also belong to the endocannabinoid system. Endocannabinoids and their analogues are produced “on demand” in the brain, liver, adipose tissue, skeletal muscle, and pancreas [3]. Among other actions, they can exert either pro- or anti-inflammatory actions and modulate the immune response [4]. Interestingly, the endocannabinoid system seems to be involved in gut physiology [5].

The gastrointestinal tract is colonized by microbial communities called gut microbiota [6], which play a relevant role in regulating the innate and adaptive immune system, gut motility, gut barrier homeostasis, nutrient absorption and fat distribution [7]. Dysbiosis, defined as an imbalance in microbial communities [8], can be induced by multiple factors, such as an unhealthy diet [9] or the use of antibiotics and other drugs [10,11], and is related to obesity and non-communicable chronic diseases [12]. Dysbiosis induces gut barrier dysfunction, which leads to the translocation from the gut into the bloodstream of Gram-negative bacterial components, such as lipopolysaccharide [13]. This fact leads to metabolic endotoxemia and systemic inflammation, characterized by an increase in gut permeability [14]. Therefore, changes in the gut microbiota composition result in gut barrier dysfunction [15].

Recent evidence has shown that the endocannabinoid system can modulate the gut microbiota composition in mice models [5,16]. Conditional adipocyte-specific deficiency in mice of N-acyl phosphatidylethanolamine phospholipase D, an enzyme involved in the synthesis of N-acylethanolamines, increased body weight and fat mass, inducing gut microbiota dysbiosis [17]. Plasma levels of certain N-acylethanolamines were positively correlated with the relative abundance of fecal bacteria in middle adults [18]. Several studies have shown that the endocannabinoid system plays a role in regulating gastric secretion [5], motility [5], and gut permeability [19] by preserving or damaging the gut barrier integrity [20], by yet unknown mechanisms.

To date, there is no scientific evidence in humans on whether plasma levels of endocannabinoids and their analogues are associated with fecal microbiota genera involved in the maintenance of gut barrier integrity. This study aimed to investigate the association of plasma levels of endocannabinoids and their analogues with fecal microbiota diversity and composition in young adults.

## 2. Material and Methods

### 2.1. Study Design and Participants

This is a cross-sectional study that was conducted within the framework of the ACTIBATE study [21], an exercise-based randomized controlled trial (ClinicalTrials.gov ID: NCT02365129). The present study included baseline data of 92 healthy young adults (27 men and 65 women, age: 18–25 years old). All assessments were performed in Granada (Spain) between October and November 2016. The included participants (i) were physically inactive and had a sedentary lifestyle (less of 20 min moderate to vigorous physical activity on less of 3 days per week), (ii) had stable body weight during the last 3 months (<3 kg change), (iii) were not smokers, (iv) did not take any medication (including antibiotics) in the last 3 months, (v) did not present any acute neither chronic illness, and (vi) were not pregnant.

The participants signed an informed consent that together with study protocol were performed in accordance with the Declaration of Helsinki, as revised in 2013. Moreover, both were approved by the Human Research Ethics Committee of the University of Granada (n°924) and the Centro de Granada, CEI-Granada.

### 2.2. Anthropometry and Body Composition Assessment

We measured weight and height with a SECA scale and a stadiometer, respectively (model 799, Electronic Column Scale, Hamburg, Germany). Dual Energy X-ray Absorptiometry (DEXA, HOLOGIC, Discovery Wi, Marlborough, MA, USA) was used for determining lean mass, fat mass and visceral adipose tissue mass. Body mass index (BMI), lean mass index and fat mass index were computed as weight, lean body mass and fat body mass (kg), respectively, divided by height squared (m^2^). The fat mass percentage was determined as the body fat mass divided by the total body mass and multiplied by 100. We measured the waist circumference (cm) in the minimum perimeter, at the end of a normal expiration, with the arms of the participants were relaxed on both sides of the body. However, if the minimum perimeter could not be detected (i.e., participants with overweight/obesity), it was measured just above the umbilicus, in a horizontal plane. Waist circumference was determined as the average of two measures with a plastic tape measure.

### 2.3. Determination of Plasma Levels of Endocannabinoids and Their Analogues

Endocannabinoids (i.e., AEA, 2-AG) and their analogues including docosahexaenoylethanolamide (DHEA), dihomo-gamma-linolenoylethanolamide (DGLEA), linoleylethanolamine (LEA), alpha-linolenoylethanolamide (alpha LEA), palmitoylethanolamine (PEA), pentadecanoylethanolamide (PDEA), palmitoleoylethanolamide (POEA), oleoylethanolamine (OEA), and stearoylethanolamine (SEA), which belong to the N-acylethanolamines class, and 2-linoleoylglycerol (2-LG) and oleoylglycerol (2-OG), which belong to the acylglycerol class [22,23], as well as AA were analyzed in plasma samples using liquid chromatography-tandem mass spectrometry (LC-MS/MS). The method followed the FDA bioanalytical method validation guidelines [24].

#### 2.3.1. Sample Preparation

The sample preparation procedure was carried out on ice to prevent analyte degradation. Endocannabinoids and their analogues were extracted using liquid–liquid extraction. Prior to the extraction, 5 µL of an antioxidant solution composed of 0.4 mg/mL of butylated hydroxytoluene (BHT) and 10 µL of an internal standard solution containing the isotopically labelled analogues were added to 150 µL of thawed plasma samples. Then, 150 µL of a buffer solution composed of 0.2 M citric acid and 0.1 M disodium hydrogen phosphate at pH 4.5 were added to the samples. Subsequently, 1 mL extraction solution composed of butanol (ButOH) and methyl-tertbutylether (MTBE) in a ratio 50:50 (*v*/*v*) was added prior to agitation for 5 min using a bullet blender and centrifugation at 16,000× *g* for 10 min at 4 °C. The organic supernatant (900 µL) was collected and evaporated to dryness using a SpeedVac instrument at room temperature. The dry residues were then reconstituted in 50 µL of an ice-cold solution of methanol (MetOH) and acetonitrile (ACN) 70:30 (*v*/*v*), prior to agitation (5 min) and centrifugation at 16,000× *g* for 10 min at 4 °C. Finally, 40 µL of the supernatant was transferred into a glass vial for further liquid chromatography-tandem mass spectrometry (LC-MS/MS) analysis.

#### 2.3.2. Liquid Chromatography-Tandem Mass Spectrometry Analysis

Relative quantitation of endocannabinoids and their analogues were performed using a SCIEX QTRAP^®^ LC-ESI-MS/MS System (SCIEX, Framingham, MA, USA). The separation was performed using a BEH C18 column (50 mm × 2.1 mm, 1.7 μm) from Waters Technologies (Milford, MA, USA), maintained at 40 °C. The mobile phase was composed of 0.1% acetic acid in water (A), ACN/0.1% acetic acid in MeOH (90:10, *v*/*v*) (B), and 0.1% acetic acid in isopropanol (C). The gradient was the following: starting condition 20% B and 1% C; increase in B from 20% to 26% between 0.75 min and 0.95 min; increase in B from 26% to 34% between 0.95 min and 6 min; increase in B from 34% to 40% between 6 min and 8 min; increase in B from 40% to 54% between 8 min and 10 min; increase in B from 54% to 56% between 10 min and 12 min; increase in C from 1% to 3% between 11 min and 12 min; increase in B from 56% to 78% between 12 min and 13 min; increase in C from 3% to 6% between 12 min and 13 min; increase in B from 78% to 85% between 13 min and 14 min; increase in C from 6% to 15% between 13 min and 14 min; conditions kept for 0.5 min prior to returning to initial conditions at 14.8 min and re-equilibration for 1.2 min. The flow rate was 0.7 mL/min. The injection volume was 10 μL, preceded by an injection of 20 μL of mobile phase A as stacking solution to improve the peak shape and increase sensitivity. MS acquisition was carried out in positive mode, with the following electrospray ionization (ESI) parameters: source temperature, 600 °C; Gas 1 (nebulizer gas), 50 L/min; Gas 2 (heater gas), 50 L/min; curtain gas, 30 L/min; collision gas, medium; ion spray voltage, 5500 V. Selected reaction monitoring was used for tandem MS experiments.

#### 2.3.3. Data Pre-Processing

SCIEX OS-MQ Software was used for peak detection and integration. The ratio of the analyte peak area to the peak area of the corresponding isotopically labelled internal standard, referred to as *peak* *area ratio*, was used for further data analysis. Quality control samples were prepared using plasma samples from healthy subjects. Quality control samples were prepared simultaneously with the study samples and regularly injected throughout the sequences. The results obtained for quality control samples were used to evaluate the quality of the data, including blank effect, retention time shifts, and peak area ratios, as well as peak area ratios, and correct for between batch variations using the in-house developed mzQuality workflow (available at http://www.mzQuality.nl, (accessed on 1 February 2020)) [25]. We calculated relative standard deviations for each internal standard and analyte present in the quality control samples. Metabolites showing relative standard deviations higher than 30% on peak area ratios in quality control samples were excluded, whereas metabolites with relative standard deviations between 15% and 30% were treated with caution (Table 1). The acylglycerols are biologically present under two isomeric forms, namely, 1-AG and 2-AG, 1-LG and 2-LG, as well as 1-OG and 2-OG, respectively. For each isomer pair, a baseline separation was obtained between the two isomers with the developed LC-MS/MS method. However, the isomeric conversion due to acyl transformation of the 2-isomer into 1-isomer after sampling is a known mechanism, notably reported for 2-AG [26]. Since this conversion could not be experimentally controlled, we summed the peak areas of both isomers 1-AG and 2-AG before calculating the peak area ratio, and labeled the isomer pair “2-AG”. The same strategy was applied for the isomer pair 1-LG and 2-LG, as well as 1-OG and 2-OG, labeled “2-LG” and “2-OG”, respectively.

### 2.4. Fecal Microbiota Analyses

#### 2.4.1. Stool Collection and DNA Extraction

The participants collected a fecal sample (~50–60 g) in plastic sterile containers, which they transported in a portable cooler to the research center. Fecal samples were stored at −80 °C until the extraction of DNA. We used a QIAamp DNA Stool Mini Kit (QIAGEN, Barcelona, Spain) to extract DNA, following the manufacturer’s instructions, and incubating the samples at 95 °C to ensure lysis of both Gram-positive and Gram-negative bacteria. The quantification of DNA was performed using a NanoDrop ND1000 spectrophotometer (Thermo Fisher Scientific, Waltham, MA, USA). The ratio of absorbance at A260/280 nm and A260/230 nm was used for measuring DNA purity.

#### 2.4.2. Sequencing Analysis

DNA extracted was amplified by PCR by primer pairs, 16S Amplicon PCR Forward Primer: 5′CCTACGGGNGGCWGCAG, and 16S Amplicon PCR Reverse Primer: 5′GACTACHVGGGTATCTAATCC targeting the V3 and V4 hypervariable regions of the bacterial 16S rRNA gene [27]. We performed all PCRs using 25 µL reaction volumes, incorporating 12.5 µL 2X KAPA HiFi Hotstart ready mix (KAPA Biosystems, Woburn, MA, USA), 5 µL of each primer (1 µM) and 2.5 µL of extracted DNA (10 ng). The cycle steps were as follows: (i) denaturation at 95 °C for 3 min, (ii) 8 cycles of denaturation at 95 °C for 30 s, (iii) annealing at 55 °C for 30 s, (iv) elongation at 72 °C for 30 s, (v) final extension at 72 °C for 5 min. AMPure XP beads (Beckman Coulter, Indianapolis, IN, USA) were used to purify the 16S V3 and V4 amplicons from free primers and primer dimers. The index PCR was performed using the Nextera XT Index Kit (Illumina, San Diego, CA, USA), which attaches dual indices and Illumina sequencing adapters, on a thermal cycler using the requirements previously mentioned. Before quantification, AMPure XP beads (Beckman Coulter, Indianapolis, IN, USA) were used for purifying the pooled PCR products. The resulting amplicons were sequenced at MiSeq (Illumina, San Diego, CA, USA), using a paired-end (2 × 300 nt) Illumina MiSeq sequencing system (Illumina, San Diego, CA, USA).

#### 2.4.3. Bioinformatics Analysis

FastQ files were analyzed with the “dada2” [28] package in R software [29]. We obtained 11,659,014 paired-ends with an average of 126,728 ± 33,395 reads per sample. All samples surpassed a cut-off of 10,000 reads. Samples were resampled to an equal sequencing depth of 30,982 reads using the “phyloseq” [30] package in R [29], retrieving 11,158 phylotypes.

The taxonomic affiliation of phylotypes was assigned using the “classifier” function from the Ribosomal Database Project (RDP), based on the naive Bayesian classification [31] with a pseudo-bootstrap threshold of 80%. A total of 209 genera that belong to 16 different phyla were obtained. In order to determinate further annotation of the phylotypes to species assignments, the “seqmatch” [32] function from RDP was performed to define the discriminatory power of each sequence read; we executed annotation according to criteria previously published [33]. Microbial communities were analyzed at different taxonomic levels (phylum to species), calculating the relative abundances, which were expressed as percentages. We performed the analyses using the genera with an average relative abundance of more than 0.5%. Only phylotypes found in at least 50% of the participants were submitted to species taxonomy level assignation.

Beta and alpha diversities were estimated based on the identified microbial communities. Beta diversity shows the differences in microbial community composition between individuals, i.e., the degree to which samples differ from one another [34], whereas the alpha diversity considers the number of different phylotypes and relative abundances within a given individual [35]. The beta diversity was quantitatively measured using permutational multivariate analysis of variance (PERMANOVA) based on the Bray–Curtis [36] similarity. The alpha diversity was assessed using richness Chao, Shannon, inverse Simpson and evenness Camargo indexes with the “microbiome” [37] package using R software [29]. The Richness Chao index estimates the diversity according to the number of different phylotypes in the community [38]; the Shannon diversity increases as both the richness and the evenness of the community increase [39]; the inverse of Simpson diversity is derived from the classical Simpson diversity and indicates the richness in a community with uniform evenness [40]; the evenness Camargo index determines the equitability of phylotypes frequencies in a community [41].

### 2.5. Determination of Plasma Levels of Lipopolysaccharides

Plasma levels of lipopolysaccharide were determined by a chromogenic limulus amebocyte lysate (LAL) assay according to the manufacturer guidelines (LAL Chromogenic Endpoint Assay; HycultBiotech, Uden, The Netherlands) as previously reported [42]. With the purpose of inactivating endotoxin neutralizing agents, we diluted the plasma samples in pyrogen-free water and heated them to 70 °C for 10 min. All samples were measured in duplicate, and results were accepted when the intra-assay coefficient of variation was <10%. Internal recovery controls were included in the assessment.

### 2.6. Statistical Analysis

Data are presented as means ± standard deviations unless otherwise stated. All variables were tested for normality using the D’Agostino and Pearson omnibus test. Since most of the variables did not display a normal distribution, non-parametric tests were used for all analyses. It has been unequivocally demonstrated that human plasma levels of endocannabinoids and their analogues are related to adiposity markers such as BMI and visceral adipose tissue mass [43,44]. Based on that, we performed and presented all the analyses adjusting for BMI and the data from both sexes pooled together, since we did not detect any sex interaction (all *p* > 0.05). To calculate beta diversity, plasma levels of endocannabinoids and their analogues were divided into tertiles (low, intermediate and high groups) and compared using a two-way PERMANOVA with 9999 permutations for significance testing with the Paleontological Statistics (Past3) software. Partial Spearman correlations were used to investigate the correlation of plasma levels of endocannabinoids and their analogues with fecal microbiota alpha diversity and composition, using the “psych” [45] and “corrplot” [46] packages in R software. Next, we divided the participants into quartiles of plasma levels of lipopolysaccharide, and we performed partial Spearman correlations, as described above, between the plasma levels of endocannabinoids and their analogues with plasma levels of lipopolysaccharide in Q1, Q4, and the whole cohort. Kruskal–Wallis tests were performed to investigate whether there were significant differences in body composition, plasma levels of endocannabinoids and their analogues and gut microbiota composition outcomes between the different quartiles of plasma levels of lipopolysaccharide with GraphPad Prism. The level of significance was assumed at *p* < 0.05. R software (V.3.6.0; http://www.R-project.org, (accessed on 1 June 2019)) and GraphPad Prism version 8.0.0 for Windows (GraphPad Software, San Diego, CA, USA) were used for the statistical analysis and graphical plots.

## 3. Results

### 3.1. Characteristics of the Participants

Table 1 shows the descriptive characteristics of the participants. AEA and seven of its analogues (i.e., AA, LEA, alpha LEA, PEA, POEA, OEA and SEA) showed relative standard deviations values of ≤15%, whereas 2-AG and five of its analogues (i.e., DHEA, DGLEA, PDEA, 2-LG and 2-OG) showed relative standard deviations values between 15% and 30%.

### 3.2. The Plasma Levels of Endocannabinoids and Their Analogues Are Not Related to Fecal Microbiota Beta and Alpha Diversities

**Figure 1:**
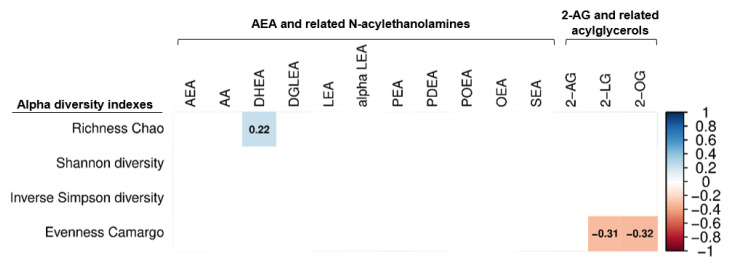
Partial Spearman correlations of plasma levels of endocannabinoids and their analogues with fecal microbiota alpha diversity indexes. Boxes only represent the statistically significant (*p* < 0.05) correlations and the value within the boxes show the spearman correlation coefficient. Blue boxes indicate positive correlations, whereas red boxes indicate negatives correlation. Body mass index was included as confounder in these correlations. 2-AG: 2-arachidonylglycerol; 2-LG: 2-linoleoylglycerol; 2-OG: 2-oleoylglycerol; alpha LEA: alpha-Linolenoyl ethanolamide; AA: arachidonic acid; AEA: anandamide; DGLEA: dihomo-gamma-linolenoyl ethanolamide; DHEA: docosahexaenoyl ethanolamide; LEA: linoleoyl ethanolamide; OEA: oleoyl ethanolamine; PEA: palmitoyl ethanolamide; PDEA: pentadecanoyl ethanolamide; POEA: palmitoleoyl ethanolamide; SEA: stearoyl ethanolamide.

The beta diversity was similar across the tertiles of the plasma endocannabinoids and their analogues at both the phylum and genus taxonomic levels (all *p* ≥ 0.312; Table 2). Overall, the plasma levels of endocannabinoids and their analogues were not related to the alpha diversity indexes (Figure 1). However, we observed that plasma levels of DHEA (related N-acylethanolamines) were positively correlated with the richness Chao index (rho = 0.23, *p* = 0.032; Figure 1), whereas plasma levels of 2-LG and 2-OG (related acylglycerols) were negatively correlated with the evenness Camargo index (rho = −0.30, *p* = 0.004, and rho = −0.31, *p* = 0.003, respectively; Figure 1). We repeated all the analyses adjusting for sex, BMI and energy intake in separate models, and the results remained the same (data not shown).

### 3.3. The Plasma Levels of Endocannabinoids and Their Analogues Levels Are Related to the Relative Abundance of Specific Fecal Microbiota Genera

**Figure 2:**
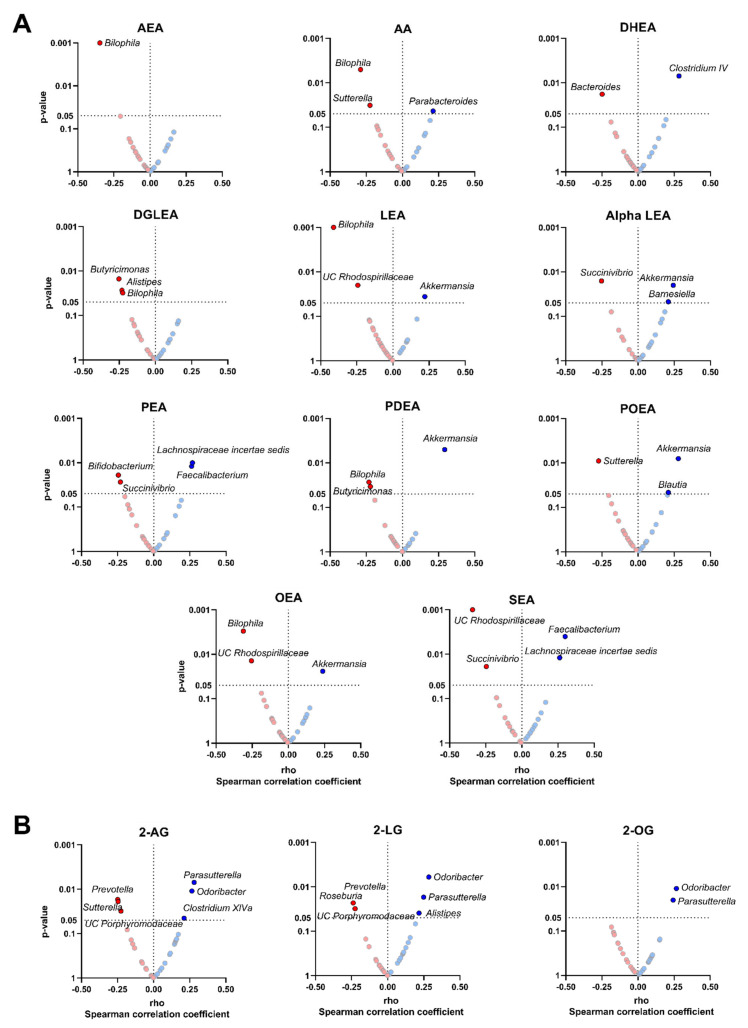
Volcano plots showing partial Spearman correlations of plasma levels of endocannabinoids and their analogues with fecal microbiota composition at genus taxonomic level. Each volcano plot shows the rho coefficients in the X-axis, whereas the *p*-values of the correlations are shown on the Y-axis. Panel (**A**) shows AEA and related N-acylethanolamines and panel (**B**) shows 2-AG and acylglycerols. Red circles indicate negative correlations, whereas blue circles indicate positive correlations. Body mass index was included as a confounder in these correlations. Only those genera with significant *p*-values (*p* < 0.05) are annotated within the figure. 2-AG: 2-arachidonylglycerol; 2-LG: 2-linoleoylglycerol; 2-OG: 2-oleoylglycerol; alpha LEA: alpha-Linolenoyl ethanolamide; AA: arachidonic acid; AEA: anandamide; DGLEA: dihomo-gamma-linolenoyl ethanolamide; DHEA: docosahexaenoyl ethanolamide; LEA: linoleoyl ethanolamide; OEA: oleoyl ethanolamine; PEA: palmitoyl ethanolamide; PDEA: pentadecanoyl ethanolamide; POEA: palmitoleoyl ethanolamide; SEA: stearoyl ethanolamide; UC: unclassified.

The plasma levels of AEA and related N-acylethanolamines, and AA were positively correlated with the relative abundance of *Verrucomicrobia* and *Firmicutes* phyla, and negatively with the relative abundance of *Proteobacteria* phylum. Similarly, the plasma levels of 2-AG and related acylglycerols were positively correlated with the relative abundance of *Proteobacteria* phylum. Specifically, we observed that the plasma levels of LEA, alpha LEA, PDEA, POEA and OEA levels were positively correlated with the relative abundance of *Akkermansia* genus (*Verrucomicrobia* phylum; all rho ≥ 0.22, *p* ≤ 0.036; Figure 2A). Additionally, the plasma levels of PEA and SEA were positively correlated with the relative abundance of *Lachnospiraceae incertae sedis* (*Firmicutes* phylum; all rho ≥ 0.26, *p* ≤ 0.010; Figure 2A) and *Faecalibacterium* genera (*Firmicutes* phylum; all rho ≥ 0.26, *p* ≤ 0.012; Figure 2A). On the opposite, the plasma levels of AEA and related N-acylethanolamines (i.e., DGLEA, LEA, PDEA and OEA), as well as AA, were negatively correlated with the relative abundance of *Bilophila* genus (*Proteobacteria* phylum; all rho ≤ −0.23, *p* ≤ 0.031; Figure 2A). Similarly, the plasma levels of alpha LEA, PEA and SEA were negatively correlated with the relative abundance of *Succinivibrio* genus (*Proteobacteria* phylum; both rho ≤ −0.23, *p* ≤ 0.027; Figure 2A). Further, the plasma levels of 2-AG and the related acylglycerols 2-LG and 2-OG were positively correlated with the relative abundance of *Parasutterella* (*Proteobacteria* phylum; all rho ≥ 0.24, *p* ≤ 0.020; Figure 2B) and *Odoribacter* genera (*Bacteroidetes* phylum; all rho ≥ 0.27, *p* ≤ 0.011; Figure 2B). In contrast, the plasma levels of 2-AG and 2-LG only were negatively correlated with the relative abundance of *Prevotella* genus (*Bacteroidetes* phylum; rho ≤ −0.24, *p* ≤ 0.023; Figure 2B). The results were similar when visceral adipose tissue mass was included as a confounder instead of BMI or physical activity levels (data not shown).

### 3.4. The Plasma Levels of Endocannabinoids and Their Analogues Are Related to the Relative Abundance of Specific Bacterial Species

**Figure 3:**
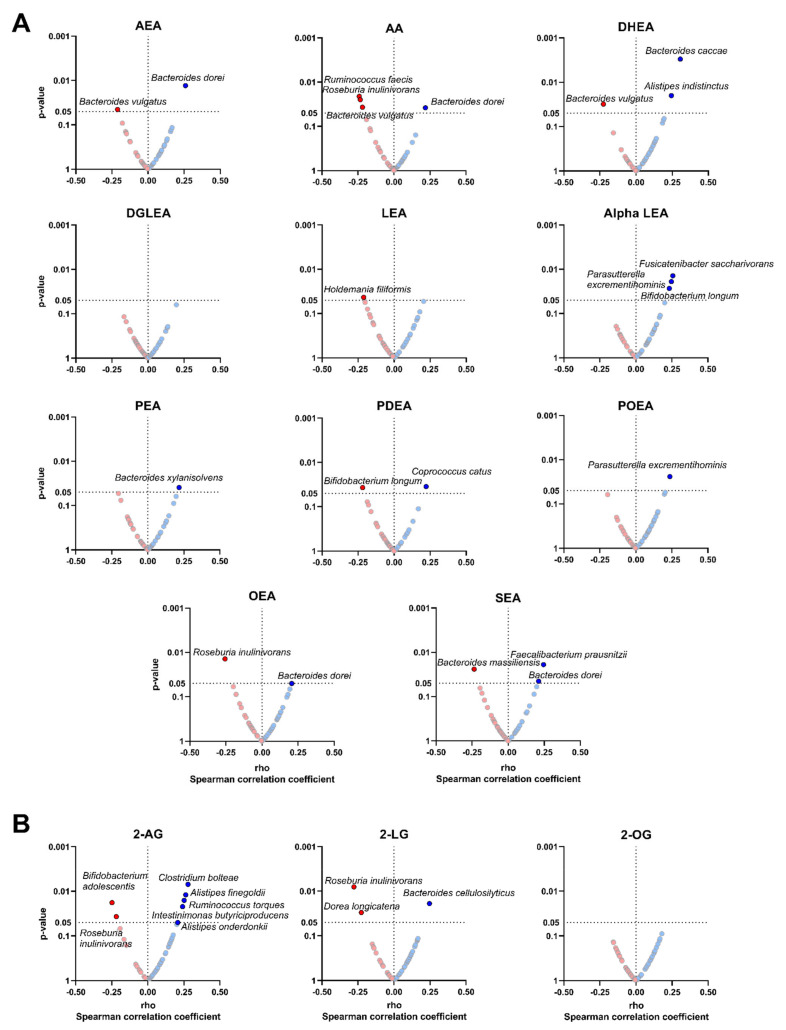
Volcano plots showing partial Spearman correlations of plasma levels of endocannabinoids and their analogues with the relative abundance of certain bacterial species. Each volcano plot shows the rho coefficients in the X-axis, and *p*-values of the correlations are shown on the Y-axis. Panel (**A**) shows AEA and related N-acylethanolamines, whereas panel (**B**) shows 2-AG and related acylglycerols. Red circles indicate negative correlations, whereas blue circles indicate positive correlations. Body mass index was included as a confounder in these correlations. Only those bacterial species with significant *p*-values (*p* < 0.05) are annotated within the figure. 2-AG: 2-arachidonylglycerol; 2-LG: 2-linoleoylglycerol; 2-OG: 2-oleoylglycerol; alpha LEA: alpha-Linolenoyl ethanolamide; AA: arachidonic acid; AEA: anandamide; DGLEA: dihomo-gamma-linolenoyl ethanolamide; DHEA: docosahexaenoyl ethanolamide; LEA: linoleoyl ethanolamide; OEA: oleoyl ethanolamine; PEA: palmitoyl ethanolamide; PDEA: pentadecanoyl ethanolamide; POEA: palmitoleoyl ethanolamide; SEA: stearoyl ethanolamide.

We investigated whether the relative abundance of specific bacterial species could drive the significant correlations observed between the plasma levels of endocannabinoids and their analogues with the relative abundance of different genera (Figure 3). We found that the plasma levels of AEA, AA, OEA and SEA were positively correlated with the relative abundance of *Bacteroides dorei* (*Bacteroidetes* phylum; all rho ≥ 0.206, *p* < 0.05; Figure 3A), whereas the plasma levels of alpha LEA and POEA were positively associated with the relative abundance of *Parasutterella excrementihominis* (*Proteobacteria* phylum; all rho ≥ 0.236, *p* ≤ 0.024; Figure 3A). Additionally, the plasma levels of SEA were positively associated with the relative abundance of *Faecalibacterium prausnitzii* (*Firmicutes* phylum; rho = 0.245, *p* = 0.019; Figure 3A). Opposite to this, the plasma levels of AEA and DHEA, as well as AA, were negatively correlated with the relative abundance of *Bacteroides vulgatus* (*Bacteroidetes* phylum; all rho ≤ −0.211, *p* ≤ 0.045; Figure 3A). Moreover, the plasma levels of AA and OEA, as well as 2-AG and the related acylglycerol 2-LG were negatively correlated with the relative abundance of *Roseburia inulinivorans* (*Firmicutes* phylum; all rho ≤ −0.219, *p* ≤ 0.037; Figure 3A,B). All associations remained significant when visceral adipose tissue mass was included as a confounder instead of BMI or physical activity levels (data not shown).

### 3.5. The Plasma Levels of Endocannabinoids and Their Analogues Are Negatively Correlated to Plasma Levels of Lipopolysaccharides but Only in Those Participants with High Plasma Levels of Lipopolysaccharides

We quantified plasma levels of lipopolysaccharide since it is an indirect marker of gut permeability [47]. We observed that plasma levels of endocannabinoids and their analogues were not correlated with the plasma levels of lipopolysaccharide (*p* ≥ 0.385; Tables S1 and S2). We then divided the cohort into quartiles of plasma levels of lipopolysaccharide: Q1 (0.14–0.35 EU/mL), Q2 (0.36–0.50 EU/mL), Q3 (0.50–1.22 EU/mL) and Q4 (1.35–5.45 EU/mL). We followed this approach because we wanted to focus on the comparison of extreme values, as well as to explore non-lineal associations. Overall, body composition, plasma levels of endocannabinoids and their analogues, and fecal microbiota composition were similar across the quartiles (Table S3). We found that plasma levels of AEA and related N-acylethanolamines (except for DHEA), as well as AA and 2-AG were positively correlated with plasma levels of lipopolysaccharide (all rho ≥ 0.22, *p* ≤ 0.035; Table 3; Table 4) only in those participants in Q1. On the opposite, plasma levels of AEA and related N-acylethanolamines (i.e., DGLEA, LEA, PEA, PDEA, POEA and OEA), as well as AA and 2-AG were negatively correlated with plasma levels of lipopolysaccharide (all rho ≤ −0.24, *p* ≤ 0.020; Table 3; Table 4) in participants in Q4. Overall, we found no correlation between the relative abundance of different genera in the feces with the plasma levels of lipopolysaccharide (Tables S4 and S5).

## 4. Discussion

The present study shows that the plasma levels of N-acylethanolamines were are positively correlated with the relative abundance of *Akkermansia* and *Faecalibacterium* genera and negatively correlated with the relative abundance of *Bilophila* genus. The plasma levels of acylglycerols were positively correlated with the relative abundance of *Parasutterella* and *Odoribacter* genera and negatively correlated with the relative abundance of *Prevotella* genus. Moreover, we observed a negative and significant correlation between the plasma levels of endocannabinoids and their analogues with the plasma levels of lipopolysaccharide, but only in participants within Q4. These findings suggest that the plasma levels of endocannabinoids and their analogues may be related in the gut barrier integrity through the gut microbiota in young adults [16]. Future studies are needed to ascertain whether endocannabinoids and their analogues could be considered markers of gut barrier integrity in humans.

### 4.1. Role of Endocannabinoids and Their Analogues in Gut Microbiota Diversity

In agreement with previous studies [48], our findings show that the beta diversity was similar across the different plasma endocannabinoids and their analogues tertiles. This could be partially explained by the homogeneity in age and cardiometabolic status of our participants (Table 1). Curiously, the plasma levels of DHEA were positive, although weakly, correlated with the richness Chao, an alpha diversity estimator, according to the number of species present in the fecal sample [38]. However, the plasma levels of 2-LG and 2-OG were negatively related to the evenness Camargo, which is a proxy of the equality in the numbers of species within the bacterial community [41]. This might indicate that an increase in the plasma levels of 2-LG and 2-OG may be related to the evenness in the bacterial community of young adults, which warrants further investigation.

### 4.2. Role of Endocannabinoids and Their Analogues in the Gut Barrier Integrity

In this study, the plasma levels of LEA, alpha LEA, PDEA, POEA and OEA were positively correlated with the relative abundance of *Akkermansia* genus. *Akkermansia*, as well as other bacteria, generates the short short-chain fatty acids acetate and propionate [49]. These short-chain fatty acids act as a primary energy source for luminal colon cells and nutrients for beneficial commensal microbial promoting gut barrier, as well as also regulating metabolic processes and energy homeostasis [49]. Interestingly, the oral supplementation of OEA for eight weeks caused a significant increase in the relative abundance of *Akkermansia muciniphila* in the colon of individuals with obesity [50]. Furthermore, higher plasma levels of the OEA/PEA ratio showed an increase in the relative abundance of *Akkermansia muciniphila* in the feces of middle-aged adults with obesity, which was associated with a reduction in systemic inflammation [18]. Interestingly, daily oral gavage administration, for 4 weeks, in the high-fat-diet-fed mice of *Akkermansia muciniphila* diet-restored the gut mucosal layer and increased gut levels of 2-AG and 2-OG, improving the gut barrier integrity and decreasing metabolic endotoxemia [51]. Therefore, our results suggest that along with OEA, LEA, alpha LEA, PDEA, and POEA could also be contributing to the preservation of the gut barrier integrity by increasing the relative abundance of *Akkermansia* genus.

Our data also show that the plasma levels of PEA and SEA were positively correlated with the relative abundance of *Faecalibacterium* genus, and the plasma level of SEA was positively related to the relative abundance of the *Faecalibacterium prausnitzii* species. *Faecalibacterium* genus and *Faecalibacterium prausnitzii* species are among the most important butyrate-producing bacteria in humans [52]. The SCFA butyrate has anti-inflammatory properties and drives the production of mucin glycoproteins [53], which promote the integrity of gut barrier [14] via secretion of glucagon-like-peptide 1 and 2 by enteroendocrine cells [54]. Similar to butyrate, the PEA present in the membrane of enteroendocrine cells is able to bind to G-protein-coupled receptor 119, reinforcing the gut barrier via secretion of glucagon-like-peptide 1 and 2. This might explain the positive association between PEA and *Faecalibacterium* genus in their protective role of the gut barrier, whereas whether it exists biological meaning of the relationship between plasma levels of SEA and this genus is unknown.

Regarding acylglycerols, we found that the plasma levels of 2-AG, 2-LG and 2-OG were positively associated with the relative abundance of *Parasutterella* and *Odoribacter* genera. Similarly, plasma levels of 2-LG were positively associated with the relative abundance of *Parasutterella* genus in mice fed with high-fat and high-sucrose diets [55]. *Parasutterella* genus influences positively the gut barrier homeostasis, via the production of short-chain fatty acids and hypoxanthine [56], a purine that improves cellular energetics and cytoskeletal function [57]. Furthermore, an increase in the relative abundance of *Odoribacter* genus was linked to an anti-obesity phenotype within the gut in Zucker rats fed with standard-chow diet, via an increase in butyrate production [58]. Altogether, there seems to exist a biological link between endocannabinoids and their analogues with short-chain fatty acids producing bacteria, mediated by actions on host intestinal cells, due to the lack of endocannabinoid receptors in bacteria [59]. However, whether this mechanism can explain how endocannabinoids and their analogues preserve gut barrier integrity should be further investigated.

It is well known that an increasing in plasma levels of lipopolysaccharide is favored by a deterioration of the gut barrier [47]. For that reason, lipopolysaccharide could be considered as a surrogated marker of gut permeability [47]. Lipopolysaccharide is released by gut microbes; moreover, individuals with metabolic syndrome or type 2 diabetes present higher circulating levels of lipopolysaccharide in comparison with healthy people [60]. In our study, plasma levels of AEA, DGLEA, LEA, PEA, PDEA, POEA, OEA, AA, and 2-AG were negatively correlated with the plasma levels of lipopolysaccharide only in participants with high lipopolysaccharide values (Q4). Of note is that when we repeated these analyses in the group of very low lipopolysaccharide values (Q1), we found opposite directions in these associations. These results suggest that when gut permeability is altered, endocannabinoids and their analogues may be related in the preservation of the gut barrier integrity. This could be explained by the low plasma levels of endocannabinoids and their analogues in participants with higher lipopolysaccharide levels, indicating a possible mobilization of endocannabinoids from the bloodstream to the intestine to reinforce the gut barrier. However, it has been shown that lipopolysaccharide might alter the gene expression of the key enzymes involved in the synthesis of endocannabinoids (N-acyl phosphatidylethanolamine phospholipase D and fatty acid amide hydrolase), and thus, lipopolysaccharide might also modulate plasma levels of endocannabinoids and their analogues [61]. Therefore, it is necessary mechanistic studies to elucidate the role of lipopolysaccharide in the endocannabinoid system synthesis.

### 4.3. Limitations and Strengths

The present study suffers the limitation of its cross-sectional design, which precludes the establishment of cause-effect relationships. Moreover, sample size was not calculated a priori, and thus, this study follows a hypothesis generation approach. The study population only included young adults, which does not enable extrapolation of results to older, younger, or unhealthy populations. Moreover, the associations observed were relatively weak. The strengths of this study are that we sequenced using the latest technology (Illumina platform) and annotated with RDP at the level of species taxon. Furthermore, this study is one of the few studies to date that have studied the relationship between endocannabinoids and their analogues with fecal microbiota diversity and composition in young adults.

## 5. Conclusions

The plasma levels of LEA, alpha LEA, PDEA, POEA, OEA, PEA, SEA, 2-AG, 2-LG, and 2-OG were positively correlated with the relative abundance of *Akkermansia*, *Faecalibacterium*, *Parasutterella* and/or *Odoribacter* genera, which may contribute to the preservation of the gut barrier integrity. Moreover, the plasma levels AEA and related N-acylethanolamines (i.e., DGLEA, LEA, PEA, PDEA, POEA and OEA), as well as AA and 2-AG, were negatively correlated with the plasma levels of lipopolysaccharide, but only in participants with high levels of lipopolysaccharide. These findings suggest that the plasma levels of endocannabinoids and their analogues are related to specific fecal bacteria genera and may play a role in the gut barrier integrity in young adults.

## Figures and Tables

**Table 1:** Descriptive characteristics of the participants.

	N	Mean	±	SD
Sex (women, %)	92	71%		
Age (years)	92	22	±	2
*Anthropometry and body composition assessment*				
Body mass index (kg/m^2^)	90	24.87	±	4.72
Lean mass (kg)	83	41.13	±	8.98
Fat mass (kg)	83	24.91	±	9.09
Lean mass index (kg/m^2^)	83	14.43	±	2.24
Fat mass index (kg/m^2^)	83	8.84	±	3.14
Fat mass percentage (%)	83	35.94	±	7.88
Visceral adipose tissue (g)	83	321.05	±	177.19
Waist circumference (cm)	90	80.60	±	13.97
*Plasma levels of endocannabinoids and their analogues (peak area ratio)*				
*AEA and related N-acylethanolamines*				
AEA	88	0.14	±	0.06
AA	88	65.18	±	21.75
DHEA *	88	0.08	±	0.16
DGLEA *	87	0.02	±	0.01
LEA	88	0.22	±	0.09
alpha LEA	88	0.008	±	0.004
PEA	87	1.78	±	0.28
PDEA *	88	0.03	±	0.01
POEA	88	0.28	±	0.22
OEA	88	0.70	±	0.21
SEA	88	1.33	±	0.23
*2-AG and related acylglycerols*				
2-AG *	88	0.02	±	0.09
2-LG *	88	0.22	±	1.41
2-OG *	88	0.01	±	0.06
*Fecal microbiota parameters*				
*Alpha diversity indexes*				
Richness Chao	92	430.68	±	150.99
Shannon diversity	92	4.21	±	0.37
Inverse Simpson diversity	92	34.20	±	14.16
Evenness Camargo	92	0.24	±	0.05
*Composition (phylum)*				
*Actinobacteria* (%)	92	1.59	±	1.55
*Bacteroidetes* (%)	92	39.80	±	9.06
*Firmicutes* (%)	92	48.65	±	10.10
*Proteobacteria* (%)	92	6.81	±	5.29
*Verrucomicrobia* (%)	92	2.09	±	4.12
*Plasma levels of lipopolysaccharide (EU/mL)*	85	0.98	±	1.10

Data are presented as means ± standard deviations (SD). 2-AG: 2-arachidonylglycerol; 2-LG: 2-linoleoylglycerol; 2-OG: 2-oleoylglycerol; alpha LEA: alpha-Linolenoyl ethanolamide; AA: arachidonic acid; AEA: anandamide; DGLEA: dihomo-gamma-linolenoyl ethanolamide; DHEA: docosahexaenoyl ethanolamide; EU: endotoxins units; LEA: linoleoyl ethanolamide; OEA: oleoyl ethanolamine; PEA: palmitoyl ethanolamide; PDEA: pentadecanoyl ethanolamide; POEA: palmitoleoyl ethanolamide; SEA: stearoyl ethanolamide. * Analytes to be considered with caution as relative standard deviations between 15% and 30% in quality control samples.

**Table 2:** Beta diversity across different tertiles of endocannabinoids and their analogues at phylum and genus taxonomic levels.

Tertiles of the Plasma Levels of Endocannabinoids and Their Analogues (Peak Area Ratio)	Phylum		Genus	
	*Pseudo-F*	*p-Value*	*Pseudo-F*	*p-Value*
*AEA and related N-acylethanolamines*				
AEA	−6.626	0.383	−6.789	0.336
AA	−5.918	0.348	−6.275	0.397
DHEA	−6.581	0.548	−6.620	0.684
DGLEA	−6.339	0.657	−6.146	0.579
LEA	−6.469	0.405	−6.670	0.418
alpha LEA	−6.211	0.361	−6.854	0.746
PEA	−6.032	0.591	−5.996	0.723
PDEA	−5.818	0.312	−6.370	0.485
POEA	−6.495	0.423	−6.516	0.336
OEA	−6.410	0.372	−6.645	0.369
SEA	−6.460	0.373	−6.797	0.442
*2-AG and related acylglycerols*				
2-AG	−6.281	0.640	−5.808	0.473
2-LG	−6.449	0.570	−6.326	0.595
2-OG	−7.041	0.578	−6.927	0.629

PERMANOVA using 9999 permutations for significance testing (*p*-value < 0.05), adjusted for body mass index. 2-AG: 2-arachidonylglycerol; 2-LG: 2-linoleoylglycerol; 2-OG: 2-oleoylglycerol; alpha LEA: alpha-Linolenoyl ethanolamide; AA: arachidonic acid; AEA: anandamide; DGLEA: dihomo-gamma-linolenoyl ethanolamide; DHEA: docosahexaenoyl ethanolamide; LEA: linoleoyl ethanolamide; OEA: oleoyl ethanolamine; PEA: palmitoyl ethanolamide; PDEA: pentadecanoyl ethanolamide; POEA: palmitoleoyl ethanolamide; Pseudo-F: statistic, larger number indicates greater separation between tertiles of plasma levels of endocannbinoids and their analogues; SEA: stearoyl ethanolamide.

**Table 3:** Partial Spearman correlation of plasma levels of AEA and related N-acylethanolamines with plasma levels of lipopolysaccharide in participants who showed very low and high values (EU/mL) adjusted for body mass index.

	AEA and Related N-acylethanolamines (Peak Area Ratio)																					
	AEA		AA		DHEA		DGLEA		LEA		Alpha LEA		PEA		PDEA		POEA		OEA		SEA	
	rho	*p*	rho	*p*	rho	*p*	rho	*p*	rho	*p*	rho	*p*	rho	*p*	rho	*p*	rho	*p*	rho	*p*	rho	*p*
Q1: Low LPS (N = 19)	0.262	**0.012**	0.228	**0.030**	0.123	0.246	0.221	**0.035**	0.404	**<0.001**	0.428	**<0.001**	0.451	**<0.001**	0.234	**0.026**	0.467	**<0.001**	0.440	**<0.001**	0.428	**<0.001**
Q4: High LPS (N = 21)	−0.464	**<0.001**	−0.389	**<0.001**	−0.001	0.992	−0.472	**<0.001**	−0.300	**0.004**	0.107	0.315	−0.304	**0.003**	−0.281	**0.007**	−0.504	**<0.001**	−0.408	**<0.001**	−0.156	0.139

Correlation analyses were performed adjusting for body mass index. Spearman’s correlations coefficient (rho) and *p*-values (*p*) obtained from univariate Spearman correlation analyses are provided. The bold indicates *p*-values < 0.05. Alpha LEA: alpha-Linolenoyl ethanolamide; AA: arachidonic acid; AEA: anandamide; DGLEA: dihomo-gamma-linolenoyl ethanolamide; DHEA: docosahexaenoyl ethanolamide; EU: endotoxins units; LEA: linoleoyl ethanolamide; LPS: lipopolysaccharide; OEA: oleoyl ethanolamine; PEA: palmitoyl ethanolamide; PDEA: pentadecanoyl ethanolamide; POEA: palmitoleoyl ethanolamide; Q: quartile; SEA: stearoyl ethanolamide.

**Table 4:** Partial Spearman correlation of plasma levels of 2-AG and related acylglycerols with plasma levels of lipopolysaccharide in participants who showed very low and high values (EU/mL) adjusted for body mass index.

	2-AG and Related Acylglycerols (Peak Area Ratio)					
	2-AG		2-LG		2-OG	
	rho	*p*	rho	*p*	rho	*p*
Q1: Low LPS (N = 19)	0.374	**<0.001**	0.111	0.295	0.171	0.106
Q4: High LPS (N = 21)	−0.243	**0.020**	0.312	**0.003**	0.053	0.615

Correlation analyses were performed adjusting for body mass index. Spearman’s correlations coefficient (rho) and *p*-values (*p*) obtained from univariate Spearman correlation analyses are provided. The bold indicates *p*-values < 0.05. 2-AG: 2-arachidonylglycerol; 2-LG: 2-linoleoylglycerol; 2-OG: 2-oleoylglycerol; EU: endotoxins units; LPS: lipopolysaccharide; Q: quartile.

## Data Availability

The data that support the findings of this study are available from the corresponding author upon reasonable request.

## Supplementary Materials

The following supporting information can be downloaded at: https://www.mdpi.com/article/10.3390/nu14102143/s1, Table S1: Partial Spearman correlation of plasma levels of AEA and related N-acylethanolamines with plasma levels of lipopolysaccharide adjusted for body mass index. Table S2: Partial Spearman correlation of plasma levels of 2-AG and related acylglycerols with plasma levels of lipopolysaccharide adjusted for body mass index. Table S3: Characteristics of the individuals according to quartiles of plasma levels of lipopolysaccharide. Table S4: Spearman correlation of relative abundance of fecal microbiota composition at genera taxonomic level with plasma levels of lipopolysaccharide. Table S5: Spearman correlation of relative abundance of fecal microbiota composition at genera taxonomic level with plasma levels of lipopolysaccharide in participants who showed very low and high values (EU/mL).

## Abbreviations

2-AG	2-arachidonoylglycerol;
2-LG	2-linoleoylglycerol;
2-OG	2-oleoylglycerol;
AA	arachidonic acid;
AEA	anandamide;
alpha LEA	alpha-Linolenoyl ethanolamide;
DGLEA	dihomo-gamma-linolenoyl ethanolamide;
DHEA	docosahexaenoyl ethanolamide;
LEA	linoleoyl ethanolamide;
NAEs	N-acylethanolamines;
OEA	oleoyl ethanolamine;
PDEA	pentadecanoyl ethanolamide;
PEA	palmitoyl ethanolamide;
POEA	palmitoleoyl ethanolamide;
SEA	stearoyl ethanolamide;
UC	unclassified.
