# Supplementary material for: Plasma Levels of Endocannabinoids and Their Analogues Are Related to Specific Fecal Bacterial Genera in Young Adults: Role in Gut Barrier Integrity
Source: Nutrients. 2022 May 20;14(10):2143. doi: 10.3390/nu14102143 (PMC9143287; doi:10.3390/nu14102143)
Supplement: Supplementary file 1 [file nutrients-14-02143-s001.zip › Table_S1.pdf]

Table S1. Partial Spearman correlation of plasma levels of AEA and related N-acylethanolamines with plasma levels of lipopolysaccharide adjusted for body mass index.

	AEA and related N-acylethanolamines (peak area ratio)																					
	AEA		AA		DHEA		DGLEA		LEA		Alpha LEA		PEA		PDEA		POEA		OEA		SEA	
	rho	P	rho	P	rho	P	rho	P	rho	P	rho	P	rho	P	rho	P	rho	P	rho	P	rho	P
LPS N=85 (EU/mL)	-0.035	0.740	-0.001	0.989	0.050	0.635	0.039	0.716	-0.092	0.385	-0.089	0.401	0.038	0.718	-0.040	0.704	-0.018	0.867	-0.057	0.594	-0.067	0.526

Correlation analyses were performed adjusting for body mass index. Spearman's correlations coefficient (rho) and P-values (P) obtained from univariate Spearman correlation analyses are provided. alpha LEA: alpha-Linolenoyl ethanolamide; AA: arachidonic acid; AEA: anandamide; DGLEA: dihomo-gamma-linolenoyl ethanolamide; DHEA: docosahexaenoyl ethanolamide; EU: endotoxins units; LEA: linoleoyl ethanolamide; LPS: lipopolysaccharide; OEA: oleoyl ethanolamine; PEA: palmitoyl ethanolamide; PDEA: pentadecanoyl ethanolamide; POEA: palmitoleoyl ethanolamide; SEA: stearoyl ethanolamide.
