# Supplementary material for: Plasma Levels of Endocannabinoids and Their Analogues Are Related to Specific Fecal Bacterial Genera in Young Adults: Role in Gut Barrier Integrity
Source: Nutrients. 2022 May 20;14(10):2143. doi: 10.3390/nu14102143 (PMC9143287; doi:10.3390/nu14102143)
Supplement: Supplementary file 1 [file nutrients-14-02143-s001.zip › Table_S2.pdf]

Table S2. Partial Spearman correlation of plasma levels of 2-AG and related acylglycerols with plasma levels of lipopolysaccharide adjusted for body mass index.

	2-AG and related acylglycerols (peak area ratio)					
	2-AG		2-LG		2-OG	
	rho	P	rho	P	rho	P
LPS N=85 (EU/mL)	0.032	0.765	-0.005	0.966	-0.060	0.575

Correlation analyses were performed adjusting for body mass index. Spearman's correlations coefficient (rho) and P-values (P) obtained from univariate Spearman correlation analyses are provided. 2-AG: 2-arachidonylglycerol; 2-LG: 2-linoleoylglycerol; 2-OG: 2-oleoylglycerol; EU: endotoxins units; LPS: lipopolysaccharide.
