# Supplementary material for: Plasma Levels of Endocannabinoids and Their Analogues Are Related to Specific Fecal Bacterial Genera in Young Adults: Role in Gut Barrier Integrity
Source: Nutrients. 2022 May 20;14(10):2143. doi: 10.3390/nu14102143 (PMC9143287; doi:10.3390/nu14102143)
Supplement: Supplementary file 1 [file nutrients-14-02143-s001.zip › Table_S3.pdf]

Table S3. Characteristics of the individuals according to quartiles of plasma levels of lipopolysaccharide.

	Quartiles of plasma levels of LPS												
	Q1 (0.14-0.35 EU/mL) n=19			Q2 (0.36-0.50 EU/mL) n=23			Q3 (0.50-1.22 EU/mL) n=22			Q4 (1.35-5.45 EU/mL) n=21			p-value
Age (years)	22	±	2	22	±	2	22	±	2	22	±	3	0.95
Sex, N													0.24
Men	3			5			7			9			
Women	16			18			15			12			
Body composition parameters													
BMI (kg/m^2)	23.54	±	5.34	24.08	±	4.27	25.73	±	5.11	26.45	±	4.14	0.05
FMI (kg/m^2)	8.29	±	3.89	8.69	±	2.83	9.03	±	3.07	9.80	±	2.75	0.33
LMI (kg/m^2)	13.94	±	1.89	13.90	±	2.03	15.13	±	2.60	14.79	±	2.51	0.25
Plasma levels of endocannabinoids and their analogues (peak area ratio)													
AEA and related N-acylethanolamines													
AEA	0.15	±	0.09	0.13	±	0.05	0.14	±	0.04	0.14	±	0.05	0.69
AA	69.30	±	19.65	64.24	±	21.88	67.53	±	27.45	66.62	±	19.62	0.95
DHEA	0.07	±	0.03	0.12	±	0.31	0.06	±	0.02	0.07	±	0.03	0.62
DGLEA	0.02	±	0.01	0.02	±	0.01	0.02	±	0.01	0.03	±	0.01	0.35
LEA	0.25	±	0.15	0.20	±	0.06	0.22	±	0.07	0.21	±	0.07	0.77
alpha LEA	0.009	±	0.007	0.008	±	0.004	0.007	±	0.003	0.006	±	0.001	0.75
PEA	1.76	±	0.32	1.71	±	0.21	1.81	±	0.28	1.76	±	0.22	0.64
PDEA	0.024	±	0.009	0.024	±	0.007	0.025	±	0.009	0.024	±	0.007	0.99
POEA	0.33	±	0.36	0.24	±	0.16	0.28	±	0.19	0.25	±	0.16	0.84
OEA	0.74	±	0.25	0.62	±	0.16	0.70	±	0.21	0.69	±	0.18	0.43
SEA	1.36	±	0.29	1.23	±	0.17*	1.42	±	0.23*	1.27	±	0.22	**0.04**
2-AG and acylglycerols													
2-AG	0.008	±	0.007*	0.048	±	0.180	0.028	±	0.032*†	0.008	±	0.006†	**0.01**
2-LG	0.03	±	0.05	0.62	±	2.73	0.18	±	0.44	0.01	±	0.02	0.07
2-OG	0.002	±	0.006	0.025	±	0.106	0.012	±	0.034	0.001	±	0.001	0.06
Fecal microbiota composition (genera, %)													
Bilophila	0.41	±	0.25	0.51	±	0.54	0.53	±	0.36	0.51	±	0.42	0.80
Akkermansia	3.20	±	4.67	2.75	±	5.56	1.64	±	2.72	1.18	±	2.95	0.30
Faecalibacterium	5.71	±	3.15	6.68	±	4.69	6.11	±	4.06	5.42	±	2.62	0.90
Parasutterella	0.72	±	1.16	2.18	±	3.20	1.28	±	2.46	0.46	±	0.70	0.35

Odoribacter	0.72	±	0.46	0.79	±	0.58	1.14	±	0.70	0.62	±	0.28	**0.04**
Prevotella	5.17	±	6.60	3.01	±	8.00	4.25	±	7.81	8.11	±	9.87	0.23

Data are presented as means ± standard deviations. Symbols * and † indicate significant differences after correcting for multiple comparisons FDR. 2-AG: 2-arachidonylglycerol; 2-LG: 2-linoleoylglycerol; 2-OG: 2-oleoylglycerol; alpha LEA: alpha-Linolenoyl ethanolamide; AA: arachidonic acid; AEA: anandamide; BMI: body mass index; DGLEA: dihomo-gamma-linolenoyl ethanolamide; DHEA: docosahexaenoyl ethanolamide; EU: endotoxins units; FMI: fat mass index; HDL-C: high-density lipoprotein cholesterol; LDL-C: low-density lipoprotein cholesterol; LEA: linoleoyl ethanolamide; LMI: Lean mass index; LPS: lipopolysaccharide; OEA: oleoyl ethanolamine; PEA: palmitoyl ethanolamide; PDEA: pentadecanoyl ethanolamide; POEA: palmitoleoyl ethanolamide; Q: quartiles; SEA: stearoyl ethanolamide.
