# Supplementary material for: Plasma Levels of Endocannabinoids and Their Analogues Are Related to Specific Fecal Bacterial Genera in Young Adults: Role in Gut Barrier Integrity
Source: Nutrients. 2022 May 20;14(10):2143. doi: 10.3390/nu14102143 (PMC9143287; doi:10.3390/nu14102143)
Supplement: Supplementary file 1 [file nutrients-14-02143-s001.zip › Table_S4.pdf]

Table S4. Spearman correlation of relative abundance of fecal microbiota composition at genera taxonomic level with plasma levels of lipopolysaccharide.

	Genera (%)											
	Bilophila		*Akkermansia*		*Faecalibacterium*		*Parasutterella*		*Odoribacter*		*Prevotella*	
	rho	P	rho	P	rho	P	rho	P	rho	P	rho	P
LPS N=85 (EU/mL)	0.021	0.851	-0.153	0.162	-0.044	0.692	-0.085	0.439	0.043	0.697	0.046	0.675

Spearman's correlations coefficient (rho) and P-values (P) obtained from univariate Spearman correlation analyses are provided. EU: endotoxins units; LPS: lipopolysaccharide.
