# Supplementary material for: Plasma Levels of Endocannabinoids and Their Analogues Are Related to Specific Fecal Bacterial Genera in Young Adults: Role in Gut Barrier Integrity
Source: Nutrients. 2022 May 20;14(10):2143. doi: 10.3390/nu14102143 (PMC9143287; doi:10.3390/nu14102143)
Supplement: Supplementary file 1 [file nutrients-14-02143-s001.zip › Table_S5.pdf]

Table S5. Spearman correlation of relative abundance of fecal microbiota composition at genera taxonomic level with plasma levels of lipopolysaccharide in participants who showed very low and high values (EU/mL).

	Genera (%)											
	Bilophila		*Akkermansia*		*Faecalibacterium*		*Parasutterella*		*Odoribacter*		*Prevotella*	
	rho	P	rho	P	rho	P	rho	P	rho	P	rho	P
Q1: Low LPS (N=19)	-0.679	**≤0.001**	0.320	0.181	0.244	0.313	0.070	0.775	0.236	0.330	-0.278	0.249
Q4: High LPS (N=21)	-0.201	0.383	0.036	0.878	-0.315	0.164	-0.126	0.586	-0.263	0.250	0.003	0.989

Spearman's correlations coefficient (rho) and P-values (P) obtained from univariate Spearman correlation analyses are provided. EU: endotoxins units; LPS: lipopolysaccharide; Q: quartile.
